# Effects of introducing routinely ultrasound scanning during Ante Natal Care (ANC) clinics on number of visits of ANC and facility delivery: a cohort study

**DOI:** 10.1186/s13690-015-0086-8

**Published:** 2015-09-07

**Authors:** Selemani Mbuyita, Robert Tillya, Ritha Godfrey, Iddajovana Kinyonge, Josephine Shaban, Godfrey Mbaruku

**Affiliations:** Ifakara Health Institute, P.O. Box 78373, Kiko Avenue, Mikocheni, Dar Es Salaam Tanzania

**Keywords:** ANC, Attendance, Vscan, Ultrasound, Facility delivery

## Abstract

**Background:**

Many countries have integrated antenatal care as an essential part of routine maternal health services. The importance of this service cannot be overemphasized as many women’s lives are usually saved particularly through early detection of pregnancy related complications. However, while many women would attend at least one visit for ante natal care (ANC), completion of recommended number of visits (4+) has been a challenge of many health systems particularly in developing countries like Tanzania.

**Methods:**

We conducted a cohort study to include ultrasound scanning using a portable hand-held Vscan to test whether by integrating it in routine ANC clinics at dispensary and health centre levels would promote number of ANC visits by women.

Health providers rendering ANC services in selected facilities were trained on how to use the simple technology of ultrasound scanning. Women living in catchment areas of the respective selected facilities were eligible to inclusion to the study when consented. A baseline status of the ANC attendance in the study area was established through baseline household and facility surveys. A total of 257 women consented and received the study treatment.

**Results:**

Our results showed that, there was no a slight change between baseline (97.2 %) and endline (97.4 %) results among women attending ANC clinics at least once. However, there was a significant change in percentage of women attending ANC clinic four times or more (27.2 % during baseline and 60.3 %; *p* = 0001).

**Conclusions:**

We conclude that, introduction of the simplified ultrasound scanning technology at lowest levels of care has an effect to improving ANC attendance in terms of number of visits and motivate facility delivery.

## Background

It is well acknowledged and known globally that early antenatal care (ANC) is important for early detection of pregnancy related complications and ultimately effective birth plan leading to better pregnancy outcomes [1]. WHO recommends a minimum of four visits in the period of pregnancy of a woman and in many cases when this is done, chances of women experiencing obstetric complications are reduced significantly [2].

**Fig. 1:**
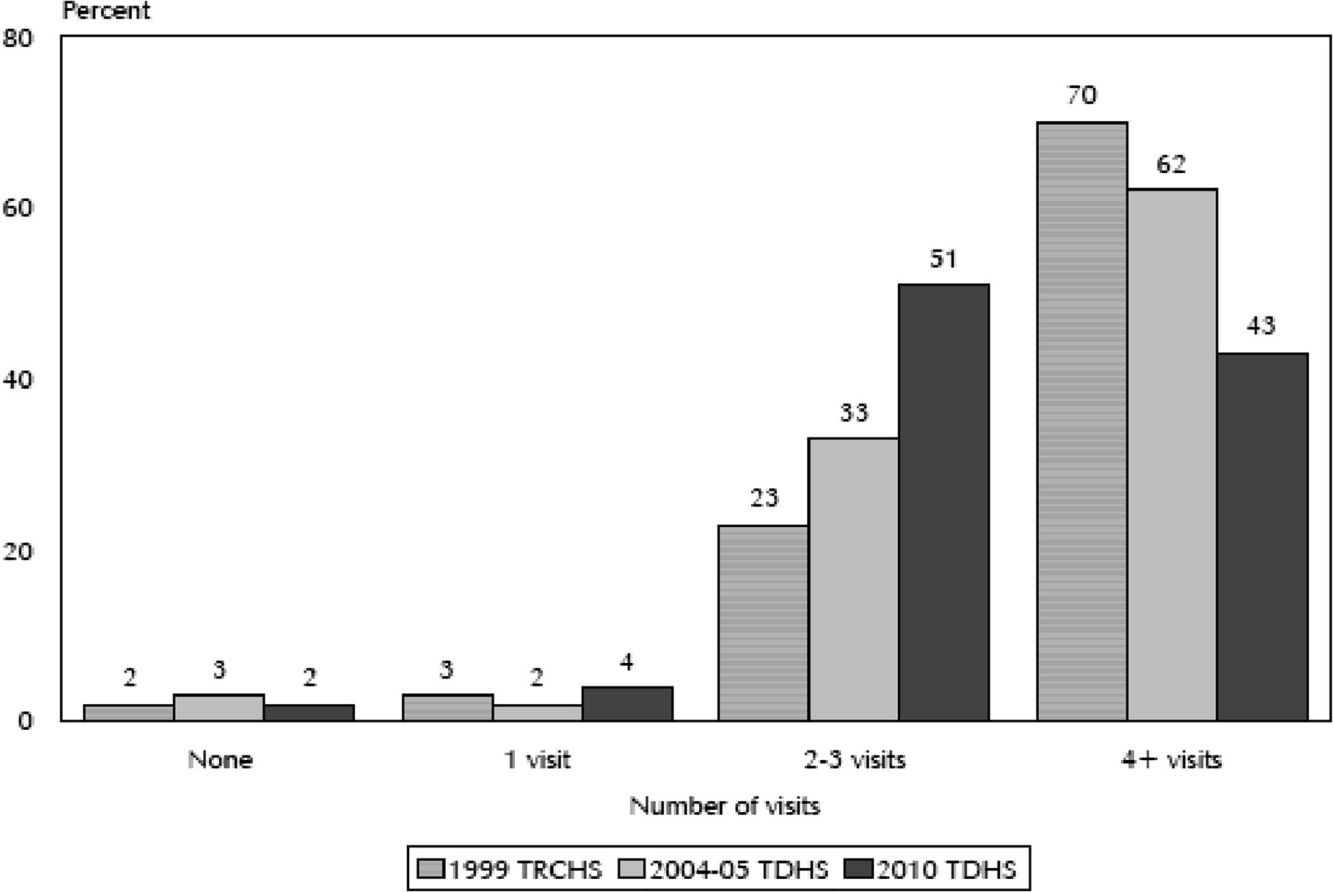
Trend of ANC attendance by Tanzanian women between 1999 and 2010 *Source*: NBS, 2010

Many developing countries (including Tanzania) have made in their health policy ANC services to be universal and provided without a cost in order to enable accessibility by the majority poor [3, 4]. However, despite such initiatives and efforts, utilization of ANC services has not been very satisfactory [5]. For example, while Tanzania has attained an average of about 96 % of women attending ANC clinics (at least once) between 2004 and 2010 (Fig. 1), the percentage of women attending four or more visits has been declining [2].

A similar trend is also observed among women who make use of health facilities during delivery. While 47 % of women delivered in health facilities in year 2004, the increase in five years later was only 3 % amounting to only 50 % of all women who gave birth in 2010 [1, 2].

Many studies have been conducted to determine barriers towards these two important indicators followed by a number of interventions but improvement has been minimal and in some cases a negative change has been realized [6, 7].

We conducted a study to test whether by introducing a simple technology of Vscan – a portable handheld ultrasound device in routine ANC services can motivate women to attend ANC clinics and complete the four or more visits. The idea is born by the fact that some studies assessing health seeking behaviour especially among rural communities found that people value and prefer technological physical examinations to just being verbally told the progress of their health [8].

The goal of this study was to contribute to the Government efforts in reducing maternal mortality and newborn deaths in order to accelerate attainment of MDG 4 and 5 using simple and appropriate technologies in rural settings of Tanzania. It aimed at assessing and evaluating acceptability, feasibility, impact and cost-effectiveness of the introduced simple technologies on maternal and newborn health.

The primary objective was to measure whether the use of ultrasound would increase the number of ANC visits by pregnant women at the dispensary level.

## Methods

### Study type and intervention

This was a cohort study applying a before and after design. The study was a Health Facility Based intervention that included clinical service improvement through clinical guidelines and pathways that were used by health care providers, a supportive and structured education and clinical leadership training and workforce development of Non-Physician Clinicians, and implementing appropriate technologies at different levels of care which included those shown in Table 1.

**Table 1:** Set of medical technologies implemented in the study

Hospital Level	Health Centre Level	Dispensary Level
Venue 40 Portable ultrasound- 1 unit. Used by physicians at highest referral level in a district health system.	Venue 40 Portable ultrasound – 1 unit. Used by Assistant Medical Officers trained by the project in ultrasound scanning at the first referral level in district health system.	Vscan Handheld Portable ultrasound – 5 units. Used by Mid-level providers (clinical officers, nurse midwives and Medical Attendants) trained by the project in ultrasound.

### Mechanisms for data collection

A baseline survey at the beginning of the project and a follow up survey at the end of the project were conducted and used to establish the project effect on quality of care on maternal and neonatal health among users (in intervention and control arms) and health providers (in interventional arm). Structured questionnaires which were adapted from Averting Maternal Death and Disability (AMDD) were used to conduct both the baseline and follow up surveys. While the structured questionnaire enabled collection of primary quantitative data, a semi-structured tool was also developed for key informant interviews to collect qualitative data which would interpret some of the quantitative data collected. Special registers were developed to capture data from pregnant women receiving the intervention.

### Study area and population

This study was conducted in Kisarawe district, one of six rural districts in the Coast Region of Tanzania. The district is third in least populated among the districts in the region (Table 2). At the time of implementing this study, the population in the district had increased to 230,456 from that indicated in Table 2. About 41 % of this population were women of reproductive age. According to district own projections, 10 % of all women who are at reproductive age were likely to become pregnant during the one year project life hence being the project targeted sub-population. Facility delivery in the district was at 73.1 % with 74 % of all deliveries attended by skilled personnel. The district has 1 hospital, 3 health centers and 20 dispensaries. Ten health facilities (and hence ten communities within catchment areas of these facilities) were selected of which five were used as intervention facilities while the remaining five were used as control facilities. In each arm, four dispensaries and one health centre were selected. The primary targeted audience was women of reproductive age who would benefit with the introduction of ultra sound scanning in routine antenatal clinic at primary facility level.

**Table 2:** Population of Pwani Region by Sex, Average Household Size and Sex Ratio

Serial No.	District/Council	Population (Number)			Average Household Size	Sex Ratio
		Total	Male	Female		
	Total	1,098,668	537,826	560,842	4.3	96
1	Bagamoyo District Council	311,740	154,198	157,542	4.4	98
2	Kibaha District Council	70,209	34,515	35,694	4.1	97
3	Kisarawe District Council	101,598	50,631	50,967	3.9	99
4	Mkuranga District Council	222,921	108,024	114,897	4.3	94
5	Rufiji District Council	217,274	104,851	112,423	4.4	93
6	Mafia District Council	46,438	22,954	23,484	3.9	98
7	Kibaha Town Council	123,488	62,653	65,835	4.1	95

*Source*: National Bureau of Statistics

### Inclusion and exclusion criteria

Only women who were attending routine ANC were included. In order to standardise follow up results as an outcome of the interventions, recruitment of women to the study considered only women who started ANC in the first trimester of their pregnancy. Also, only women whose residences were within the selected health facility catchment area were involved. The age of targeted women was limited to 15–49 years. Pregnant women who use the facility through other inlets such as out patient department, emergency section, labour room or normal admission benefited from the interventions for ethical reasons but were not included in the study. A sample size of 257 women was pre-determined using STATA computer software.

### Duration of the treatment

The study population received the interventions three times (during the first, second and third trimesters of the pregnancy). Only when clinically required, the client received an extra treatment of the interventions. The treatment was specifically provided from the 6^th^ week (recruitment and first measurement) to 34^th^ week of gestation. The project lasted for one year.

### Statistical analysis

Analysis of data generated from training was generally descriptive, with frequency distribution of the respondents (pregnant women) across variables calculated. For each arm of the study (intervention and control), data from household surveys involving women who accessed ANC services within the catchment areas of the respective health facilities were developed into two separate data sets and analysed separately. Then the percentage change in the level of knowledge and confidence in assessing pregnancy related complications among health providers was derived by comparing the before and after intervention figures assessed during facility assessments. Normal frequency distribution across most of the variables was calculated and two-sided paired *t*-test used to generate statistical significance. The before and after figures were used to arrive into conclusion if changes have occurred in both arms while comparisons of results from the two arms was used to associate changes with interventions.

### Research ethics

This work received ethical approval from the Ifakara Health Institute – Institutional Review Board (IHI – IRB) - certificate number IHI/IRB/No.35. The IRB is registered with FEDERAL WIDE ASSURANCE NUMBER: SWA 00002632.

## Results

The primary indicator of the study was to determine whether by introducing ultrasound scanning in routine ANC services would increase the number of visits by women attending ANC clinics. While there was no a significant change between baseline and endline results among women attending ANC clinics at least once (97.2 % during baseline and 97.4 % during endline), there was a significant change in percentage of women attending ANC clinic four times or more. Similarly, facility delivery increased significantly (Table 3).

**Table 3:** Change in key indicators as a result of introducing routine ultrasound scanning in ANC clinics (%)

Indicator	Intervention area			Control area		
	Baseline (n-381)	Endline (423)	p-value	Baseline (n-394)	Endline (383)	p-value
ANC visits (at least once)	97.2	97.4	0.0412	96.9	96.1	0.3002
ANC Visits (four times and above)	27.2	60.3	0.0001	32.4	34.1	0.0891
Facility delivery	79.2	87.9	0.002	67.7	69.4	0.0623

**Fig. 2:**
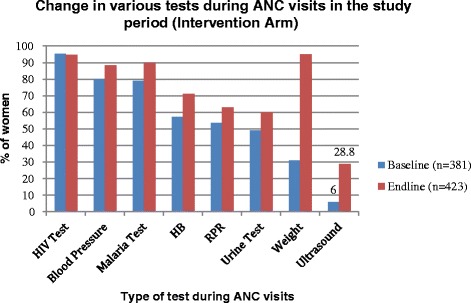
Improvement on availability of ultrasound scanning during ANC clinic in the study interventional arm

On the other hand, percentage of women who had access to ultrasound services increased significantly (Fig. 2). Before the study was introduced, the only facility where such services were available was the district hospital. More than 66 % (*n* =381) of the women from the study area used to traveling between 5 and 78 km to the district hospital to access ultrasound services when referred to by health providers from lower health facilities.

The results from the study also demonstrated increase (Tables 4 and 5) in confidence of mid-level providers in the study facilities in determining pregnancy related complications (*p* = 0.0001).

**Table 4:** Confidence of health providers in detecting pregnancy related complications (%) [Baseline; n = 30]

	Anteparturm hemorrhage	Transverse lie	Placenta previa	Breech presentation	Ectopic pregnancy	Twins	Poly /oligo hydromnious
Very confident	23.3	13.3	10	13.3	3.3	13.3	0
Confident	50	60	23.3	56.7	16.7	60	33.3
Not confident	10	13.3	46.7	23.3	40	27.3	43.3
Cannot measure/diagnose	16.7	13.3	20	6.7	40	0	23.3

**Table 5:** Confidence of health providers in detecting pregnancy related complications [End-line; n = 32]

	Anteparturm hemorrhage	Transverse lie	Placenta previa	Breech presentation	Ectopic pregnancy	Twins	Poly/oligo hydromnious
Very confident	29.03	45.16	25.81	41.94	19.35	25.81	25.81
Confident	67.74	41.94	41.94	51.61	29.03	54.84	29.03
Not confident	3.23	9.68	19.35	6.45	32.16	12.9	29.03
Cannot measure/diagnose	-	3.23	12.9	-	19.35	6.45	16.13

The results include also increase in referrals made to higher facilities from the study dispensaries. During baseline, only 148 obstetric referrals were made to the higher facilities (based on clinical guidelines of the ministry of health rather than actual detection of pregnancy related complications). At endline, a total of 765 referrals were made (comprising both those according to clinical guidelines and detected using the Vscan) after introducing the Vscan in the district. Although there was no baseline data for compliance of referral before the study started, the study realized 78 % compliance among women who were referred to higher facilities.

## Discussion

Results from this study add to the body of knowledge currently available on strategies and interventions that can boost improvement in ANC utilization. While many interventions had been tied to the socio-cultural and health system factors [6, 9] the technological angle has not received a significant attention in this area [10]. Some studies have tried to improve ANC attendance through incentives such as cash and other physical goods and materials. In other places in Tanzania, locally inverted enforcement mechanisms such as fining a partner whose wife/partner has not attended one or all of ANC clinics have been tried but with less success or if successful then not sustainable [Mbuyita *et al.* 2007, unpublished observations].

Improvements in technology such as the Vscan are recent scientific positive resolutions that offer alternative solutions in public health [11]. In fact, the reproductive health sector has delayed to take advantage of this technology compared to the cardiology sector where quite a number of studies have been conducted using the device in detecting and managing cardiovascular related problems in rural settings where sophisticated machines are unavailable [12].

The simplicity of the Vscan in its use makes the training to health providers and its application being simple. It also offers a motivational tool to health providers working in rural clinics by building up their confidence [13]. In addition, the technology helps the health providers (majority of whom are less skilled) to make rational and correct decision [14] on the proper management of the pregnant women and or provide referral [15].

This study intends to also provide cost analysis of this intervention which we optimistically perceive to be cheap based on the available data (to be analysed) and when compared to the benefits of the pregnancy outcomes. In studies conducted in Asia, the use of the device was able to help 89 % of women at high risk of complicated delivery change their birth plan after being scanned and complications detected [16]. The improved ability of the rural ANC clinics in which majority of the women access ANC services will translate into reduced traveling distances hence less indirect cost in accessing maternal health services in addition to improved pregnancy outcomes [17] whose value cannot be compared in monetary terms.

Finally, compliance to referrals has been reported by several studies to be a problem in maternal health. Because of the traveling cost, lack of accommodation and food of the people accompanying the referred woman, long distances to the referral facilities and perceived loss of household income when bread winners have to accompany the woman to a referral facility, many women have not complied to referrals given. The high percentage of women who complied to the referral provided to them in the study area suggest that women and their families need a convincing evidence to make them believe that there is a need for a referral. Such convincing power can be offered by visual images from ultrasound scanning shown to the family.

## Conclusions

This study concludes that, the introduction of the Vscan for routine ultrasound scanning as part of integrated ANC services has an effect to motivating women to attend ANC clinics four times or more. It also has an effect to making women comply to referrals provided in cases where complications were detected and in turn motivate women to deliver in health facilities.

## Abbreviations

ANC: Antenatal Care
IHI: Ifakara Health Institute
IRB: Institutional Review Board
MDG: Millennium Development Goals
TDHS: Tanzania Demographic Health Surveillance
